# Price's equation made clear

**DOI:** 10.1098/rstb.2019.0361

**Published:** 2020-03-09

**Authors:** Andy Gardner

**Affiliations:** School of Biology, University of St Andrews, Greenside Place, St Andrews KY16 9TH, UK

**Keywords:** covariance, George R. Price, least-squares regression, Price equation, Price's theorem, social evolution

## Abstract

Price's equation provides a very simple—and very general—encapsulation of evolutionary change. It forms the mathematical foundations of several topics in evolutionary biology, and has also been applied outwith evolutionary biology to a wide range of other scientific disciplines. However, the equation's combination of simplicity and generality has led to a number of misapprehensions as to what it is saying and how it is supposed to be used. Here, I give a simple account of what Price's equation is, how it is derived, what it is saying and why this is useful. In particular, I suggest that Price's equation is useful not primarily as a predictor of evolutionary change but because it provides a general theory of selection. As an illustration, I discuss some of the insights Price's equation has brought to the study of social evolution.

This article is part of the theme issue ‘Fifty years of the Price equation’.

## 1. Introduction

George R. Price (1922–1975) was a restless, obsessive thinker. His eclectic career saw him working as a chemist on the Manhattan Project and at Bell Laboratories, writing about economics and extra-sensory perception as a science journalist, developing mainframe computing at IBM, pursuing evolutionary theory at the Galton Laboratory, undertaking biblical exegesis in relation to the Easter story and becoming consumed by fundamentalist Christianity—before his life was tragically cut short by suicide [1–3]. During his time in London, Price made several major contributions to evolutionary theory [4], including the first application of game theory to animal behaviour [5], a reinterpretation and proof of R. A. Fisher's fundamental theorem of natural selection [6] and the derivation of the equation that now bears his name [7,8].

Price's equation provides a very simple—and very general—encapsulation of evolutionary change. Though first employed to capture the action of natural selection in the context of evolutionary genetics [7], Price subsequently generalized it to encompass all forms of evolutionary change, with a diversity of possible applications to biological and non-biological domains [8,9]. Price's equation now provides the formal underpinnings of several topics in evolutionary biology, particularly in relation to the evolution of social behaviour [10,11], and it has found applications across a wide range of different disciplines, from evolutionary biology to epidemiology [12] to community ecology [13] to cosmology [14] to information theory [15].

However, the combination of simplicity and generality that gives Price's equation its power has also given rise to a range of misapprehensions as to what the equation actually states and how it is properly employed. This is understandable. Price's equation is unusual in how it emerges from purely notational definitions, rather than from the kind of mechanical assumptions that more conventionally provide the building blocks of mathematical models. Consequently—at some level—it does not tell us anything that we did not already know.

Here, I aim to provide a simple account of what Price's equation is, how it is derived, what it is saying and why it is useful. On the way, I hope to dispel certain confusions that have arisen as to its generality and its scientific value. In particular, I will suggest that Price's equation is useful not primarily as a predictor of evolutionary change but because it underpins a general theory of selection, and I will make clear that although the equation has a deep connection with linear regression, it does not in and of itself rest upon any linearity assumption. I will provide a concrete illustration of the conceptual usefulness of Price's equation by considering some of the insights it has brought to the study of social evolution.

## 2. Price's equation

Price's equation is a statement about the difference between two assemblages. In its evolutionary biology applications, the assemblages are usually two successive generations of a biological population, so that the difference between them describes how that population becomes transformed across a single generation of evolutionary change. However, in other applications, Price's equation might be describing differences between non-biological assemblages, and these differences might be occurring through space or other dimensions, rather than necessarily through time.

More specifically, Price's equation describes the change in the average value of some character of interest that occurs between the two assemblages. The average value of the character among the entities that make up the first assemblage—which are usually termed the ‘parents’—is denoted *E_i_*_∈_*_I_* (*z_i_*), where *z_i_* is the character value of parent *i* and *I* is the set of all parents. And the average value of the character among the entities that make up the second assemblage—which are usually termed the ‘offspring’—is denoted *E_i_*_∈_*_I_* (*z_i_*′), where *z_i_*′ is the character value ascribed to the offspring of parent *i*. Accordingly, the difference in the average value of the character between parent and offspring assemblages is Δ*E_i_*_∈_*_I_* (*z_i_*) = *E_i_*_∈_*_I_* (*z_i_*′) – *E_i_*_∈_*_I_* (*z_i_*), and Price's equation states that this difference is given by

2.1 $$\Delta E_{i\in I}\;(z_{i})=\text{Co}{\text{v}}_{i\in I}(w_{i},z_{i})+E_{i\in I}(w_{i}\Delta z_{i})$$

where *w_i_* is the relative contribution made by parent *i* to the offspring assemblage (this parent's ‘fitness’) and $\Delta z_{i}=z_{i}^{\prime }-z_{i}$ is the difference in character value between parent *i* and its offspring (see box 1 for a full derivation).

Box 1. **Derivation of Price's equation.**

Price's equation emerges from a mapping between two aggregates, termed ‘parents’ and ‘offspring’, respectively [4]. To make this mapping, I first assign each parent a unique identifier; in the example below, each parent is assigned a number, so that I am able to refer to ‘individual 1’, ‘individual 2’ and so on up to ‘individual 5’. More generally, I assign each parent a unique index *i* ∈ *I*, where *i* refers generically to an individual's index and *I* is the set of all indices that have been assigned. 
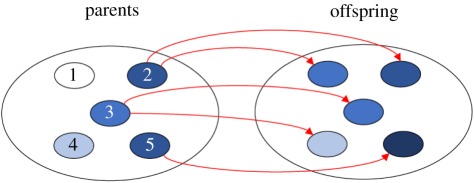

Next, I assign each parent a relative abundance *q_i_*, denoting the extent to which they make up the population. In many applications, the relative abundance is simply *q_i_* = 1/*N* for each of the *N* individuals existing in parental aggregate (i.e. *q_i_* = 1/5 in the above example), but more generally one might wish to assign some of the individuals more weight than others because, for instance, they might be physically larger. The important constraint here is that all the *q_i_* values, as proportions, are constrained to take values between zero and one (i.e. 0 < *q_i_* ≤ 1 for all *i* ∈ *I*), and they are also constrained to sum to one (i.e. ∑*_i_*_∈*I*_ *q_i_* = 1). I then focus on a particular characteristic of these individuals, denoted by *z_i_*. This can be any quantity that takes a real numerical value, representing any characteristic from the size of the individual's antlers to the individual's proclivity to altruism. In the above example, it is represented by shading.

Turning to the offspring aggregate, I now map each offspring to one and only one parent, and I notate this mapping by assigning each offspring the same index as its parent. In biological applications, one might have in mind asexual reproduction where each organism is descended from a single parent. However, the formalism readily extends to sexual reproduction, for example, if the entities in the offspring aggregate are thought of as successful gametes that have been produced by individuals in the parental aggregate. I then notate the relative abundance of the offspring of individual *i* as *q*_i_′ and this, too, is constrained by its definition as a proportion (i.e. $0\leq q_{i}^{\prime }\leq 1$ for all *i* ∈ *I* and ∑*_i_*_∈*I*_ $q_{i}^{\prime }=1$). The fitness of each parent entity can then be defined as the relative growth of its lineage, $w_{i}=q_{i}^{\prime }/q_{i}$ ; note that this constrains all fitnesses to be non-negative (i.e. *w_i_* ≥ 0 for all *i* ∈ *I*) and to be one on average (i.e. ∑*_i_*_∈*I*_ *q_i_ w_i_* = ∑*_i_*_∈*I*_ *q_i_* $(q_{i}^{\prime }/q_{i})=$∑*_i_*_∈*I*_ $q_{i}^{\prime }=1)$. Finally, I notate the character value of the offspring of parent *i* by $z_{i}^{\prime }=z_{i}+\Delta z_{i}$, where Δ*z_i_* simply describes how parent and offspring differ and we impose no constraints on whether this quantity is positive or negative or zero. Where a parent has multiple offspring that vary in their character value, one can interpret *z_i_*′ as an average taken over the brood.

With these definitions in place, the difference between the average trait value of the parent and offspring aggregates is

$$\begin{array}{rl} \Delta E_{i\in I}(z_{i})=\; & E_{i\in I}({z^{\prime }}_{i})-E_{i\in I}\;(z_{i})=\underset{i\in I}{\sum }{q^{\prime }}_{i}{z^{\prime }}_{i}-\underset{i\in I}{\sum }q_{i}z_{i}=\underset{i\in I}{\sum }q_{i}w_{i}(z_{i}+\Delta z_{i})-\underset{i\in I}{\sum }q_{i}z_{i}=\underset{i\in I}{\sum }q_{i}w_{i}z_{i}-\underset{i\in I}{\sum }q_{i}z_{i}+\underset{i\in I}{\sum }q_{i}w_{i}\Delta z_{i}\\ & \;=E_{i\in I}\;(w_{i}z_{i})-E_{i\in I}\;(w_{i})E_{i\in I}\;(z_{i})+E_{i\in I}\;(w_{i}\Delta z_{i}) \end{array}$$

where, in the final line, I have made use of the fact that $E_{i\in I}\;(w_{i})=1$. Noting that $E_{i\in I}\;(w_{i}z_{i})-E_{i\in I}\;(w_{i})E_{i\in I}\;(z_{i})=\text{Co}{\text{v}}_{i\in I}\;(w_{i},z_{i})$, I arrive at equation (2.1) of the main text

$$\Delta E_{i\in I}\;(z_{i})=\text{Co}{\text{v}}_{i\in I}\;(w_{i},z_{i})+E_{i\in I}\;(w_{i}\Delta z_{i}).$$

Price's equation expresses overall evolutionary change as the sum of a ‘selection’ term Δ*_S_E_i_*_∈_*_I_*(*z_i_*) ≡ Cov*_i_*_∈_*_I_*(*w_i_*,*z_i_*) and a ‘transmission’ term Δ_T_*E_i_*_∈_*_I_*(*z_i_*) ≡ *E_i_*_∈_*_I_*(*w_i_*Δ*z_i_*). These terms provide very general definitions of the concepts of selection and transmission that apply across a very wide domain, including the biological and the non-biological, and to evolutionary changes occurring through time as well as to other kinds of population transformation that occur across space and other dimensions.

In many applications, it is desirable to express the action of selection as an expectation over future uncertainty as to how the offspring aggregation will be constituted, and to thereby eliminate chance effects [16]. To implement this, I assign every possible outcome a unique index *ω* and I denote the set of all possible outcomes *Ω*. Then, the total expected change between parent and offspring aggregates is given by

$$\begin{array}{rl} E_{\omega \in \Omega }\; & (\Delta E_{i\in I}{\left(z_{i}\right)}^{\omega })=\;E_{\omega \in \Omega }(\text{Co}{\text{v}}_{i\in I}(w_{i}^{\omega },z_{i}))+E_{\omega \in \Omega }(E_{i\in I}(w_{i}^{\omega }\Delta z_{i}^{\omega }))=\text{Co}{\text{v}}_{i\in I}(E_{\omega \in \Omega }(w_{i}^{\omega }),z_{i})+E_{\omega \in \Omega }(E_{i\in I}(w_{i}^{\omega }\Delta z_{i}^{\omega })), \end{array}$$

which recovers Δ_S_*E_i_*_∈_*_I_* (*z_i_*) ≡ Cov*_i_*_∈_*_I_* (*w_i_*,*z_i_*) so long as we understand fitness $w_{i}=E_{\omega \in \Omega }(w_{i}^{\omega })$ to represent an expectation with respect to future uncertainty, rather than a measure of reproductive success realized under any particular outcome [16].

Of particular interest is the application of Price's equation to the concept of natural selection [7,17,18]. This is a particular kind of selection, in which the unit of selection (notated by *i*) is a biological organism, the arena of selection (notated by *I*) is a population of such organisms, the character under selection (notated by *z*) is the heritable component of an organism's phenotype (given by a weighted sum of the alleles carried by the individual; [7]) and the target of selection (notated by *w*) is the organism's Darwinian fitness (see [19] for more discussion).

That is, Price's equation says that the change in the average character value between parent and offspring assemblages is equal to the sum of two quantities. The first quantity is the covariance of fitness and character value across parents, and this defines the action of *selection*. Selection favours an increase in the trait insofar as it is associated with greater fitness. The second quantity is the average of the product of fitness and the character difference between parent and offspring, and this defines the non-selective *transmission* portion of evolutionary change. Transmission effects contribute to evolutionary change, interfering with the action of selection, when there are systematic differences between parents and their offspring.

## 3. Vacuous or profound?

Price's equation is a very general result, on account of the way that it follows directly from definitions and hence is relatively lacking in generality-limiting assumptions. The equation emerges from rearrangement of notation rather than, say, from physical laws, so it is not a prediction of the change that occurs between the two aggregates but rather a mathematical identity that shows one way in which such change can be expressed. Some have argued that this makes the equation useless, in the sense that it does not tell us anything we did not already know. However, I believe that this view arises from a misunderstanding of how Price's equation is actually employed.

There is an old football joke, repeated by footballers down the ages when they are asked how their team will ensure that they win the next game. Their answer: ‘by scoring more goals than the other team’. Van Veelen *et al.* [20] have likened Price's equation to this quip, saying that although the equation is correct it is vacuous as it does not tell us anything we did not already know. However, the pertinence of the answer depends greatly on context. If a child is learning how to play football, she might reasonably ask how her team is to go about winning the game. And the answer to her question is: ‘by scoring more goals than the other team’. Far from being vacuous, the information conveyed in this answer is absolutely crucial if one wants to know how to win a game of football. Scoring more goals than the other team is literally the aim of the game.

Price's equation is saying something similar. It is not making a substantive *prediction* about what population change will occur in any particular context; this is something that would require a more mechanistic model and a suite of generality-limiting assumptions. Instead, Price's equation is *defining* evolutionary change—or, more properly, its component parts. Importantly, Price's equation provides a completely general, formal definition of selection. This is something that no mechanical model that makes particular, generality-limiting assumptions is able to do—a mathematical model may instantiate and exemplify selection, but it cannot define selection unless the model assumptions apply as generally as the concept of selection itself.

## 4. A theory of selection

Price's equation highlights that there are two conceptual components to evolution, namely selection and transmission. It makes no *a priori* claim that either of these two components has quantitative primacy over the other, but there is a sense in which Price's equation places greater conceptual importance upon the action of selection. This is because Price's equation emerges from the key constraint of conservation of frequency [15]: in a strong sense, Price's equation describes evolutionary change as that which owes to changes in the frequencies of things (i.e. selection) and that which does not (i.e. everything else, collected under the umbrella-term ‘transmission’). That is, first and foremost, Price's equation provides a general theory of selection, and insofar as it does also define transmission, this is so as to carefully remove non-selective effects from consideration.

This point highlights that while Price's equation captures the action of selection in terms of the mathematics of covariance, it involves a rather special kind of covariance, which we might term a ‘selection covariance’. Put another way, although Price's equation suggests that every kind of selection can be represented as a covariance, not every covariance admits a selection interpretation. Specifically, while covariance, in general, refers to statistical association between two random variables, a selection covariance requires that one of these random variables admits an interpretation as a fitness score—that is, a growth factor relating one proportion to another, with the usual constraints applying to those proportions (each proportion falls in the unit interval and the sum of all proportions is equal to one).

More generally, consideration of the mathematical components of a selection covariance reveals that there are four basic elements underpinning the logic of selection [19]. First, associated with any selection covariance is the concept of the *unit of selection*. This is the entity class in the parental aggregate that I have indexed by *i*. In standard evolutionary scenarios, the unit of selection is the individual organism. Second, there is the concept of the *arena of selection*. This is the set of all the units that defines the aggregate itself, which is notated by *I*. In standard evolutionary scenarios, the arena of selection is a biological population. Third, there is the concept of the *character under selection*. This is the character of interest notated as *z* in the selection covariance, and in evolutionary scenarios, this is often the genetical portion of an organismal phenotype. Fourth, there is the concept of the *target of selection*, notated as *w*. This is the quantity whose covariance with the character drives the action of selection, and in evolutionary scenarios, it defines the concept of fitness.

Price's encapsulation of the action of selection differs strikingly from how selection is more usually captured in population genetics models. The difference in approach arises from a difference in aims. With population genetics models, the aim is usually to provide a prediction of evolutionary change, under the action of natural selection and other non-Darwinian forces, across a number of generations. To give the model solidity, the population geneticist focuses on that which is unchanging, i.e. the allele or genotype, and describes how the frequencies of the different alleles and genotypes change over the course of evolution. In contrast with this typological approach, Price's equation typically pushes alleles and genotypes into the background (perhaps leaving them implicit, or even dispensing with these concepts altogether) and instead focuses attention on the individual organism and her personal idiosyncracies, formalizing the way in which natural selection favours the fittest individuals and consequently generates whole-organismal adaptations.

In other words, Price's equation represents a return to the original Darwinian logic of natural selection. This was perhaps best demonstrated by Price [7] when he showed that the selection covariance can be expressed as the product of the least-squares linear regression of fitness against character value and the variance in character value

4.1 $$\Delta_{S}E_{i\in I}\;(z_{i})=\text{Co}{\text{v}}_{i\in I}(w_{i},z_{i})=\beta_{i\in I}(w_{i},z_{i})\text{Va}{\text{r}}_{i\in I}(z_{i}),$$

as this captures Darwin's basic argument that selection occurs when there is variation in a character of interest (Var*_i_*_∈*I*_ (*z_i_*) ≠ 0) that is correlated with fitness (*β_i_*_∈*I*_(*w_i_*,*z_i_*) ≠ 0).

This link between the action of natural selection and the mathematics of least-squares linear regression—first grasped by Fisher [21]—has been much misunderstood. Some authors have interpreted it as meaning that Price's equation *assumes* linearity or additivity, and that it is therefore liable to give incorrect results in the context of more complex relationships between genes, phenotypes and fitness. However, this is incorrect; Price's equation is instead showing that natural selection only *cares* about additive effects: irrespective of the goodness of the least-squares straight-line fit, the product of the slope of this straight line and the heritable variance correctly describes the action of natural selection.

## 5. Insights for social evolution

Owing to Price's close interactions with W. D. Hamilton, the first applications of Price's equation were to the field of social evolution [8,22,23]. Here, the equation has provided major conceptual insights, particularly into the relationship between kin selection and group selection [23,24], and it now provides the formal foundations for both of these social evolutionary topics [10,11].

During the first half of the twentieth century, it was commonplace for biologists to unreflectively invoke Darwinism in order to explain the evolution of adaptations that appeared to function for the good of the species. Often, the basic assumption was that whatever phenotypes benefit the individual must also benefit the whole social group and ultimately the entire species. And many supposed that, even if a trait were to be detrimental to the individual, it would nevertheless be favoured by natural selection, provided it yielded a benefit for the species as a whole. However, during the middle years of the century, it became increasingly clear that the fitness interests of the individual can come into conflict with those of her wider social group. This spurred theoretical attention into the evolution of altruistic behaviour, in which the individual sacrifices some or all of her own reproductive success in order to improve the reproductive success of her social partners.

Two competing explanations for altruism soon emerged, both having their roots in Darwin's own work. First, the *kin selection* explanation suggested that altruism can be favoured by natural selection, despite the fitness cost to the altruist, provided that this leads to a sufficiently large benefit for her relatives, who will tend to share copies of the altruism allele and hence pass it onto subsequent generations via their own reproductive success [25–27]. Second, the *group selection* explanation suggested that altruism can be favoured, despite placing the altruist at a disadvantage relative to the rest of her social group, provided that it yields a large enough benefit for the group as a whole [27,28]. The rest of the century saw a heated debate between these two viewpoints, with theoreticians scrambling to develop population genetical models of kin selection and group selection to evaluate which was likely to be the most important driver of altruism in the natural world [29].

Price's equation has been identified as largely resolving this debate, by showing that the kin selection and group selection approaches to social evolution are actually just different ways of describing the very same thing. The key to establishing their mathematical equivalence has been to provide a general encapsulation of natural selection and to show how this can be re-expressed in alternative kin selection versus group selection forms [23]. The kin selection approach takes the overall covariance between individual trait and fitness across the whole population and, using multiple regression, divides this into two separate components: the first component is the part of fitness that owes to the impact that an individual's trait has on her own fitness and the second component is the part of fitness that owes to the impact of social partners' traits on each others’ fitness, with the kin selection coefficient of relatedness emerging as a statistical regression of social partner genotypes (box 2). The group selection approach takes exactly the same overall covariance between individual trait and fitness across the whole population and, using the law of total covariance, divides this into two components in a different way: the first component is the portion of the trait–fitness covariance that occurs at the between-group level and the second component is the portion of the trait-fitness covariance that occurs at the within-group level (box 2).

Box 2. **Kin selection and group selection.**

The kin selection approach uses multiple regression to separate the overall association between an individual's genetical trait and her relative fitness into the direct effect of her own trait on her own fitness and the indirect effect of her genetically related social partner's trait on her own fitness, the latter indirect effect driving the action of kin selection [30,31]. Mathematically, this may be written as

*β_i_*_∈*I*_ (*w_i_*,*z_i_*) = *β_i_*_∈*I*_ (*w_i_*,*z_i_*|*Z_i_*) + *β_i_*_∈*I*_ (*w_i_*,*Z_i_*|*z_i_*) *β_i_*_∈*I*_ (*Z_i_*,*z_i_*)

where *β_i_*_∈*I*_ (*w_i_*,*z_i_*|*Z_i_*) = −*c* describes the partial effect of the individual's own trait value *z_i_* on her own fitness *w_i_*, holding fixed the trait value *Z_i_* of her social partner; *β_i_*_∈*I*_ (*w_i_*,*Z_i_*|*z_i_*) = *b* describes the partial effect of the individual's social partner's trait value *Z_i_* on her fitness *w_i_*, holding fixed her own trait value *z_i_*; and *β_i_*_∈*I*_ (*Z_i_*,*z_i_*) = *r* describes the statistical association between the genetical trait values of social partners, i.e. the kin selection coefficient of genetic relatedness (a graphical illustration of this multiple regression analysis is provided below). Accordingly, from equation (4.1), the condition for natural selection to favour an increase in the average level of the genetical trait is *β_i_*_∈*I*_ (*w_i_*,*z_i_*) > 0 and hence −*c* + *br* > 0, i.e. Hamilton's [22,25,26] rule of kin selection. This result pertains to the scenario in which social partners interact in relation to only one role, for example, when individuals are all directly equivalent and interact in pairs, but it readily extends to encompass social partners interacting in multiple roles [32]. 
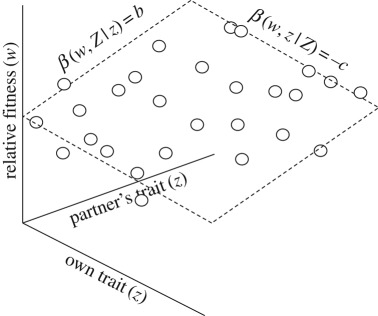

This foregoing account of kin selection has been framed in terms of the personal fitness approach, which describes how an individual's personal fitness is modulated by the phenotypes of her social partners (potentially including herself; [26]). An alternative framing of kin selection is provided by the inclusive fitness approach, which describes how an individual's phenotype modulates the fitnesses of her social partners (potentially including herself; [26]). These two approaches yield different notions of fitness at the individual level, but they describe exactly the same selective phenomenon when these fitness effects are aggregated across the entire population (see [31] for more discussion).

A different way of partitioning the overall action of natural selection is achieved by separating individuals into discrete groups and decomposing the population-level covariance of fitness and trait value into the portion that exists at the between-group level plus the portion that exists at the within-group level. This group selection approach emerges from a simple application of the law of total covariance, and expresses the overall action of natural selection as

*Δ*_S_*E_i_*_∈_*_I_*(*z_i_*) = Cov*_j_*_∈*J*_(*E_k_*_∈*K*_(*w_jk_*), *E_k_*_∈*K*_(*z_jk_*)) + *E_j_*_∈*J*_(Cov*_k_*_∈*K*_(*w_jk_*,*z_jk_*))

where I have assigned every group a unique index *j* ∈ *J* and every individual within a given group a unique index *k* ∈ *K* [8,23]. The first term in this sum describes the covariance, across all the groups in the population, of the average fitness and average trait value within each group, and this defines the action of between-group selection. The second term describes the average, across all the groups in the population, of the covariance of fitness and trait value within each group, and this defines the action of within-group selection. (An illustration of this group selection partition, in which individuals are represented by discs and group boundaries by dashing, is given below.) While the above result pertains to a simple scenario in which individuals are arranged into groups, with no further hierarchical structuring of the population, the selection covariance mathematics also readily extends to scenarios with an arbitrary number of levels of biological organization.
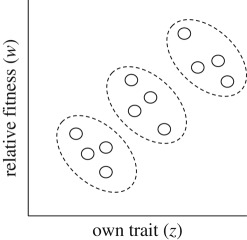

Note that the between-group portion of natural selection can be expressed as Cov*_j_*_∈*J*_ (*W_j_*, *Z_j_*), where group fitness *W_j_* = *E_k_*_∈*K*_(*w_jk_*) is defined as the average of the fitness of its constituent members, and group trait *Z_j_* = *E_k_*_∈*K*_(*z_jk_*) is defined as the average of the traits of its constituent members. That is, between-group selection is directly analogous to standard, individual-level natural selection, but with the group taking on the role of the unit of selection, the group's phenotype acting as the character under selection and group fitness being the target of selection [19]. This contrasts with an alternative, ‘contextual analysis’ approach to group selection [11,33], which defines group selection in terms of the impact of group phenotype on individual fitness, such that the individual organism remains the unit of selection and the individual's fitness remains the target of selection [34].

Owing to the way in which more-standard population genetics models tend to rest upon generality-limiting mechanistic assumptions, with the modeller aiming to develop the simplest model that captures the desired effects, the usual population genetics approach to modelling kin selection or group selection has been to build upon more basic models of natural selection by adding extra mechanistic assumptions to incorporate these evolutionary forces. This has led to a persistent misconception that kin and group selection are not part of standard natural selection, but something extra to be invoked only in special circumstances—such as when our attention turns to altruistic behaviour. By contrast, by framing selection in its full generality from the outset, Price's equation reveals that kin and group selection are components of natural selection, and we obtain their dynamics by drawing them out of—rather than adding them into—the basic form of Price's equation. Moreover, by showing how the kin selection and group selection viewpoints both emerge from the mathematics of natural selection, Price's equation shows that these are not competing hypotheses for the evolution of social behaviour but simply different ways of conceptualizing the very same evolutionary process—and that a fierce, decades-long debate had been largely over nothing. This powerfully illustrates just how useful a general theory of natural selection can be.

In addition to clarifying the logical connections between kin selection and group selection, Price's equation has also yielded fundamental insights into each of these topics separately. With regard to kin selection, perhaps the first insight was that genetic relatedness, as a statistical regression coefficient, may take negative as well as non-negative values—which means that an individual is genetically less similar to her social partner than she is, on average, to a random member of her population—and this breakthrough paved the way for a theory of the evolution of spiteful behaviour [1, p.173; 22]. More generally, Price's equation dispels a persistent misconception that Hamilton's rule assumes additivity of gene action, by showing that it emerges in a fully general form directly from the action of natural selection itself (see [31] for more discussion). Finally, Price's equation makes clear the unit of selection within the theory of kin selection: while some researchers have conflated kin selection with the ‘gene's eye view’ or have argued that it is a type of group selection in which selection operates at the level of the ‘kin group’, Price's equation makes clear that the standard framing of kin selection is as an individual-level process, driven by a covariance between individual trait and individual fitness.

Similarly, Price's equation has provided clarity to the topic of group selection, in showing that there are two distinct selection covariances at work: the first corresponding to between-group selection, with the group itself representing the unit of selection; and the second corresponding to within-group selection, with the individual representing the unit of selection. In contrast with earlier suggestions that, wherever the interest of individual and group are in conflict, it is necessarily the group's interests that prevail [28], Price's equation shows that *a priori* there is no reason to suspect that either of the two selection covariances will fully dominate the other. Moreover, while a ‘contextual analysis’ [11,33] partition of natural selection has been touted as providing an alternative theory of group selection, a Pricean examination of the elements of the corresponding selection covariance reveals that it takes the individual organism as the unit of selection and the individual's fitness as the target of selection, such that it is not capturing group selection in any traditional sense of the term [34].

## 6. Discussion

Price's equation provides a very general description of evolutionary change, but it has generated some confusion as to what it is really saying and how it is supposed to be used. Here, I have argued that its primary usefulness is in providing a general theory of selection, by isolating the Darwinian portion of evolution from the rest of evolutionary change.

Why do we need a general definition of selection? First, this definition enables us to cut to the heart of the concept of selection, stripping away features that are extraneous rather than integral to the concept. Darwin's theory of natural selection was inspired by Malthus's view that the intrinsic multiplicative nature of biological reproduction inevitably leads to intense competition for limiting resources, such that the growth of one lineage or population tends to impact negatively upon the growth of its competitors, and this resource competition has often been seen as a defining feature of natural selection. But resource competition does not feature explicitly within Price's equation, and this reveals that it is not a fundamental component of the logic of Darwinism. Indeed, natural selection occurs even when genetic lineages grow exponentially—provided that they grow at different rates.

Second, if we did not have a general formal definition of selection, then there would be potential for different researchers to be talking about quite different things when they use the term natural selection, resulting in complicated and mutually incompatible predictions. It might appear that everyone understands exactly what natural selection is, or at least sufficiently well to carry out useful biological research, but the pervasiveness of vacuous ‘for the good of the species' thinking during the first half of the twentieth century makes clear that this is by no means correct. Having a purely notational meta-model that captures all specific scenarios enables evolutionary biologists to be sure that they are talking about the same thing when they talk about natural selection.

Third, when formal definitions are lacking, it is possible for researchers to believe that they are talking about different things when they are actually talking about the same thing. With the eventual realization during the middle years of the twentieth century that natural selection does not straightforwardly work for the good of the species, a heated, decades-long debate raged over whether kin selection or group selection better explained the evolution of altruistic behaviour. Price's meta-model of natural selection, with its ready application to social effects and to selection in hierarchically structured populations, provides a means of proving that (in general, and not just in particular special cases) the kin selection and group selection approaches to natural selection simply represent different ways of describing the very same evolutionary process.

Fourth, by stripping away extraneous, context-specific details and framing selection in a more fundamental way, surprising and informative connections may be made between dramatically different topics (see [15] for an overview). Some of the connections between selection and other concepts have been sufficiently surprising to have been a source of confusion—for example, the way in which the language of selection is formally related to least-squares linear regression has confused some researchers into thinking that, in order to yield correct results, Price's equation requires that a linearity assumption be met. Arguably, *all* human understanding ultimately rests upon analogy, and so by making explicit the formal connections between different disciplines, we are better placed to take insights that have been hard-won in some domains and translate them into new understanding in quite different areas.

## Data accessibility

This article has no additional data.

## References

[1] Hamilton WD 1996 Narrow roads of gene land volume 1—evolution of social behaviour. Oxford, UK: W. H. Freeman.

[2] Schwartz J 2000 Death of an altruist. Lingua Franca 10.

[3] Harman O 2010 The price of altruism. New York, NY: W. W. Norton & Co.

[4] Frank SA 1995 George Price's contributions to evolutionary genetics. J. Theor. Biol. 175, 373–388. (10.1006/jtbi.1995.0148) 7475081

[5] Maynard Smith J, Price GR 1973 The logic of animal conflict. Nature 246, 15–18. (10.1038/246015a0)

[6] Price GR 1972 Fisher's ‘fundamental theorem’ made clear. Ann. Hum. Genet. 36, 129–140. (10.1111/j.1469-1809.1972.tb00764.x) 4656569

[7] Price GR 1970 Selection and covariance. Nature 227, 520–521. (10.1038/227520a0) 5428476

[8] Price GR 1972 Extension of covariance selection mathematics. Ann. Hum. Genet. 35, 485–490. (10.1111/j.1469-1809.1957.tb01874.x) 5073694

[9] Price GR 1995 The nature of selection. J. Theor. Biol. 175, 389–396. (10.1006/jtbi.1995.0149) 7475082

[10] Frank SA 1998 Foundations of social evolution. Princeton, NJ: Princeton University Press.

[11] Okasha S 2006 Evolution and the levels of selection. Oxford, UK: Oxford University Press.

[12] Day T, Gandon S 2005 Insights from Price's equation into evolutionary epidemiology. In Disease evolution: models, concepts and data analysis (eds Feng ZL, Dieckmann U, Levin SA), pp. 23–44. Providence, RI: American Mathematical Society.

[13] Fox JW 2006 Using the Price equation to partition the effects of biodiversity loss on ecosystem function. Ecology 87, 2687–2696. (10.1890/0012-9658(2006)87[2687:UTPETP]2.0.CO;2) 17168013

[14] Gardner A, Conlon J 2013 Cosmological natural selection and the purpose of the universe. Complexity 18, 48–56. (10.1002/cplx.21446)

[15] Frank SA 2018 The Price equation program: simple invariances unify population dynamics, thermodynamics, probability, information and inference. Entropy 20, 978 (10.3390/e20120978) PMC7512578 33266701

[16] Grafen A 2000 Developments of the Price equation and natural selection under uncertainty. Proc. R. Soc. Lond. B 266, 1223–1227. (10.1098/rspb.2000.1131) PMC1690660 10902688

[17] Darwin CR 1859 The origin of species. London, UK: John Murray.

[18] Fisher RA 1930 The genetical theory of natural selection. Oxford, UK: Clarendon Press.

[19] Gardner A 2015 The genetical theory of multilevel selection. J. Evol. Biol. 28, 305–319. (10.1111/jeb.12566) 25475922 PMC4415573

[20] Van Veelen M, Garcia J, Sabelis MW, Egas M 2012 Group selection and inclusive fitness are not equivalent; the Price equation vs. models and statistics. J. Theor. Biol. 299, 64–80. (10.1016/j.jtbi.2011.07.025) 21839750

[21] Fisher RA 1918 The correlation between relatives on the supposition of Mendelian inheritance. Trans. R. Soc. Edinb. 52, 399–433. (10.1017/S0080456800012163)

[22] Hamilton WD 1970 Selfish and spiteful behaviour in an evolutionary model. Nature 228, 1218–1220. (10.1038/2281218a0) 4395095

[23] Hamilton WD 1975 Innate social aptitudes of man: an approach from evolutionary genetics. In Biosocial anthropology (ed. Fox R), pp. 133–155. New York, NY: Wiley.

[24] Frank SA 1986 The genetic value of sons and daughters. Heredity 56, 351–354. (10.1038/hdy.1986.56) 3733459

[25] Hamilton WD 1963 The evolution of altruistic behavior. Am. Nat. 97, 354–356. (10.1086/497114)

[26] Hamilton WD 1964 The genetical evolution of social behaviour I & II. J. Theor. Biol. 7, 1–52. (10.1016/0022-5193(64)90038-4) 5875341

[27] Maynard Smith J 1964 Group selection and kin selection. Nature 201, 1145–1147. (10.1038/2011145a0)

[28] Wynne-Edwards VC 1962 Animal dispersion in relation to social behaviour. Edinburgh, UK: Oliver & Boyd.

[29] Leigh EG 2010 The group selection controversy. J. Evol. Biol. 23, 6–19. (10.1111/j.1420-9101.2009.01876.x) 20002254

[30] Queller DC 1992 Quantitative genetics, inclusive fitness and group selection. Am. Nat. 139, 540–558. (10.1086/285343)

[31] Gardner A, West SA, Wild G 2011 The genetical theory of kin selection. J. Evol. Biol. 24, 1020–1043. (10.1111/j.1420-9101.2011.02236.x) 21371156

[32] Grafen A 2006 Optimization of inclusive fitness. J. Theor. Biol. 238, 541–563. (10.1016/j.jtbi.2005.06.009) 16046225

[33] Heisler IL, Damuth J 1987 A method for analysing selection in hierarchically structured populations. Am. Nat. 130, 582–602. (10.1086/284732)

[34] Gardner A 2015 More on the genetical theory of multilevel selection. J. Evol. Biol. 28, 1747–1751. (10.1111/jeb.12684) 26264884

